# Hormetic Heat Shock Enhances Autophagy through HSF1 in Retinal Pigment Epithelium Cells

**DOI:** 10.3390/cells11111778

**Published:** 2022-05-28

**Authors:** Mooud Amirkavei, Flavia Plastino, Anders Kvanta, Kai Kaarniranta, Helder André, Ari Koskelainen

**Affiliations:** 1 Department of Neuroscience and Biomedical Engineering, School of Science, Aalto University, 00076 Espoo, Finland; mooud.amirkavei@aalto.fi; 2 Department of Clinical Neuroscience, Division of Eye and Vision, St. Erik Eye Hospital, Karolinska Institutet, 17177 Stockholm, Sweden; flavia.plastino@ki.se (F.P.); anders.kvanta@ki.se (A.K.); helder.andre@ki.se (H.A.); 3 Department of Ophthalmology, Institute of Clinical Medicine, University of Eastern Finland and Kuopio University Hospital, 70210 Kuopio, Finland; kai.kaarniranta@uef.fi

**Keywords:** autophagy-associated genes, autophagosomes, autolysosomes, heat shock response, heat shock transcription factor, homeostasis

## Abstract

To maintain homeostasis, cells have evolved stress-response pathways to cope with exogenous and endogenous stress factors. Diverse stresses at high doses may be detrimental, albeit low doses of stress, known as hormesis, can be beneficial. Upon exposure to stress, such as temperature rise, the conventional heat shock response (HSR) regulated by the heat shock transcription factor 1 (HSF1) facilitates refolding of misfolded proteins with the help of heat shock proteins (HSPs). However, the role and molecular mechanisms underlying the beneficial effects of HSR with other clearance processes, such as autophagy, remain poorly understood. In this study, human ARPE-19 cells, an in vitro model of retinal pigment epithelium, were treated with hormetic heat shock (HHS) and the autophagy expression profile was examined using quantitative PCR (qPCR), immunoblotting, immunoprecipitation, and immunofluorescence. We demonstrate that HHS enhances the expression of fundamental autophagy-associated genes in ARPE-19 cells through the activation of HSF1. HHS transiently increases the level of SQSTM1 and LC3B-II and activates autophagy. These findings reveal a role for autophagic HSF1-regulated functions and demonstrate the contribution of autophagy to hormesis in the HSR by improving proteostasis.

## 1. Introduction

The accumulation of damaged and harmful components in cells, particularly those composed of misfolded and potentially hazardous proteins, is characteristic for several neurodegenerative diseases, including Alzheimer’s disease, Parkinson’s disease, and age-related macular degeneration (AMD) [1,2,3]. Misfolded proteins frequently expose hydrophobic patches that structurally should be buried inside the protein, making the misfolded proteins prone to aggregation. Inefficient protein homeostasis and failures in removing misfolded and aggregated proteins or impaired organelles, especially damaged mitochondria, can lead to the production of reactive oxygen species and increase in oxidative stress [4]. In the eye, retinal pigment epithelial (RPE) cells are responsible for preserving the health of the retina by providing nutrients and removing waste materials, and by performing functions, such as outer blood-retinal barrier formation and phagocytosis of shed photoreceptor outer segments [5,6]. However, the increase in age-related oxidative stress and cellular waste leads to incomplete protein degradation by overloaded ubiquitin-proteasome (UPS) and autophagic systems, culminating in RPE and photoreceptor cell loss in advanced stages of AMD [7]. To maintain cellular homeostasis, cells have evolved highly regulated stress response pathways. Macroautophagy (hereafter, referred to as autophagy) is a multistep lysosome-dependent process involved in cellular homeostasis and degradation of sequestered cytoplasmic materials [8]. Under stressful conditions, such as hypoxia, nutrient starvation, and oxidative stress, the mammalian target of rapamycin (mTOR) signaling is inhibited and the Unc-51–like kinase (ULK1) is activated, leading to the initiation of autophagy [9,10,11]. Conserved autophagy proteins encoded by autophagy-related genes (*ATG*) are involved in the sequential process of autophagy [12]. Initially, the Beclin-1 (BECN1; the mammalian homolog of Atg6) complex promotes the nucleation of phagophore membranes. The phagophores expand in the elongation step; the microtubule-associated protein I light chain 3 beta (LC3B-I; the mammalian homolog of Atg8) is conjugated with phosphatidylethanolamine (PE) and converted to LC3B-II, which is inserted into the autophagosomal membrane by the action of ATG5 and ATG7 proteins [13,14,15]. The synthesis and processing of LC3-II is upregulated during activation of autophagy. LC3-II is almost solely expressed in autophagic structures, making it a key marker of autophagy in cells. Autophagy can either be non-selective, degrading randomly cytosolic components, or autophagosomes can selectively sequester specific cargos for destruction. The function of the latter is based on autophagic cargo receptor proteins that bind to and carry cytoplasmic cargos to autophagophores through binding to LC3B-II. Sequestosome-1 (SQSTM1; often also referred to as p62) has an LC3-interacting region and a binding site for ubiquitin, which makes SQSTM1 a key cargo receptor protein in mammalian cells. In the next step of the autophagic process, the cargo is surrounded by the autophagophore membrane, forming the autophagosome [16,17]. The autophagic process terminates with fusion of the autophagosomes with lysosomes to form autolysosomes, where sequestered cytoplasmic cargo and the inner membrane of the autophagosome are degraded by lysosomal hydrolases [18]. Both the lysosome-associate membrane protein 2 (LAMP2) and the Ras-related protein 7 (RAB7) play important roles in the final steps of the autophagic flux, where LAMP2 has been associated with lysosome biogenesis [19] and RAB7 regulates the fusion of autophagosomes and lysosomes into autolysosomes [18]. 

Upon a rise in temperature as a stress factor, heat shock factor 1 (HSF1), a master regulator of protein homeostasis, translocates into the nucleus, where it trimerizes to the active transcription factor and modulates the cellular heat shock response (HSR). Trimeric HSF1 binds to heat shock elements (HSE) in the promoter of target genes and induces the expression of heat shock proteins (HSP) as well as other molecular chaperons [20]. In heat shock stress, HSPs recognize unfolded or misfolded proteins and act to correct their folding or mediate their removal through the UPS or chaperone-mediated autophagy [21,22]. 

Various transpupillary fundus heating methods have been developed with the aim of treating retinal diseases, such as AMD. The emphasis of these studies has been to apply temperature increases in order to activate the HSR yet to prevent cellular damage [23,24,25]. Previous studies have demonstrated the induction of HSPs in the RPE cells after transpupillary laser irradiation [24,25,26,27,28,29], which could lead to therapeutical effects [24,25,30,31,32]. However, the molecular mechanisms underlying the beneficial effects of HHS in RPE cells have not been fully unraveled yet. On the other hand, aggravating autophagy deregulation and reduction in the autophagic flux in RPE cells from AMD patients indicates that reduced autophagy is correlated with the disease’s progression [33,34,35]. Studies of AMD are limited to available RPE cells. The human RPE cell line ARPE-19 has been used extensively as an in vitro model. Albeit, ARPE-19 does not retain all characteristics of naïve RPE cells [36], and recent studies have centered in human embryonic stem (hES) or human-induced pluripotent stem (hiPS) cell-derived RPE as alternatives to modeling AMD [37,38]. 

In this study, we examined the HSR, HSF1, and heat-induced activation of autophagy in human ARPE-19 cells. The HHS-induced HSF1 was followed by the expression of HSP70, as expected for a canonical heat shock responder. We found that HHS-induced HSF1 modulates the expression of several autophagy-associated genes, which resulted in the formation of larger autophagosomes and enhanced cargo presentation to the lysosome. These results suggest that hormetic heat shock can be used to increase autophagic flux in mammalian RPE cells and thus to potentially improve their waste clearance, which might be beneficial in AMD.

## 2. Materials and Methods

### 2.1. Cell Culture

The human retinal pigment epithelial cell line ARPE-19 was obtained from American Type Culture Collection (ATCC, CRL-2302). The cells were cultured in DMEM/F12 medium (1:1 mixture Dulbecco’s Modified Essential Medium and Ham’s F-12 Medium; ThermoFisher Scientific: Waltham, MA, USA, 31331093), supplemented with 10% fetal bovine serum (FBS; ThermoFisher Scientific, 10500064), and 1% penicillin-streptomycin antibiotics (P/S; ThermoFisher Scientific, 15140122) to reach full confluency and to form cuboid or hexagonal morphologies.

### 2.2. Hormetic Heat Shock Treatment

ARPE-19 cells were given a single bout of HHS at 42 °C for 30 min by placing the culture plates in a water bath, followed by recovery at 37 °C for 3, 12, and 24 h. Control cells were maintained at 37 °C for 24 h and not exposed to HHS (non-HHS). To evaluate the HHS impact on the autophagy flux, the cells were first pre-exposed for 6 h to 50 nM Bafilomycin A1 (Baf), an autophagy inhibitor (Sigma-Aldrich: St. Louis, MO, USA, SML1661) at 37 °C. Then, the cultures were heated and recovered as described above in the presence of Baf. Non-HHS controls in the presence of Baf were collected simultaneously with the recovery times.

### 2.3. HSF1 Gain-of-Function

The human HSF1 open-reading frame (NM_005526.4) cloned in-frame into the FLAG-tagged pcDNA3.1(+) was purchased from GenScript (Piscataway, NJ, USA). ARPE-19 cells were transfected with 2 μg pDNA encoding FLAG-HSF1 or empty FLAG backbone with lipofectamine LTX with plus reagent (ThermoFisher Scientific, 15338030), according to the manufacturer’s instructions. Cell cultures were harvested 36 h post-transfection and prepared for immunoblotting or qPCR as described below.

### 2.4. Immunoblotting

Total proteins were extracted from cells using RIPA lysis buffer (Sigma-Aldrich, 20–188) containing protease (Roche: Basel, Switzerland, 11836170001) and phosphatase (Roche, 4906837001) inhibitor cocktails. Protein extracts were quantified by a Bradford protein assay (Bio-Rad Laboratories: Hercules, CA, USA, 5000201). A total of 15 µg of total protein was separated by 4–20% stain-Free TGX SDS-PAGE gels and transferred onto polyvinylidene difluoride (PVDF) membranes (Bio-Rad Laboratories, 1620177). Then, membranes were blocked with Tris-buffered saline (TBS) with 1% casein (Bio-Rad Laboratories, 1610782) for 1 h at room temperature (RT). Subsequently, the membranes were incubated overnight (ON) at 4 °C with primary antibodies diluted in a blocking solution; anti-HSP70 (1:1000, mouse polyclonal; Santa Cruz Biotechnology: Dallas, TX, USA, SPA-810); anti-SQSTM1 (1:1000, rabbit monoclonal; Novus Biologicals: Abingdon, UK, NBP1-48320); anti-LC3B (1:1000, rabbit monoclonal; Cell Signaling Technology: Danvers, MA, USA, 3868); anti-HSF1 (1:500, rabbit monoclonal; Cell Signaling Technology, 12972); and anti-FLAG (1:500, mouse monoclonal; Sigma-Aldrich, F1804). Incubation with secondary antibodies diluted in a blocking solution was performed 1 h at RT: anti-rabbit-IgG conjugated to horseradish peroxidase (1:10,000; Dako: Carpinteria, CA, USA, P044801-2); anti-rabbit-IgG StarBright 700 (1:2500; Bio-Rad Laboratories, 12004161); anti-mouse-IgG StarBright 520 (1:2500; Bio-Rad Laboratories, 12005866); and loading control hFAB-Rhodamine anti-Actin (1:2000; Bio-Rad Laboratories, 12004164). Membranes were extensively washed with TBS (Bio-Rad Laboratories, 1706435) supplemented with 0.05% Tween-20 (Sigma-Aldrich, 9005-64-5) after all antibody steps. Finally, the protein of interest was visualized either by enhanced chemiluminescence (Clarity ECL; Bio-Rad Laboratories, 1705061) or direct fluorescence with a Chemidoc MP imaging system (Bio-Rad Laboratories, Hercules, CA, USA). Image Lab 3.0 software (Bio-Rad Laboratories, Hercules, CA, USA) was used to determine the optical density (OD) of the bands, and protein levels was corrected to the Actin as a loading control.

### 2.5. Immunoprecipitation 

The protein extracts from six independent experiments were pooled into one sample to produce a high protein content. A total of 15 μL of Dynabeads protein G (ThermoFisher Scientific, 10003D) was blocked with 1% bovine serum albumin (BSA; Sigma-Aldrich, A9418) in TBS, for 30 min at RT under rotation, followed by immunization with 5 μL of rabbit monoclonal anti-LC3B (rabbit monoclonal; Cell Signaling Technology, 3868) for 1 h at RT. After magnetizing the beads and removal of the antibody solution, 300 µg of total protein extract diluted to a final volume of 500 μL with TBS was added to the immunized beads and incubated ON at 4 °C under rotation. Following extensive washes with TBS-T, cleared protein complexes were eluted by boiling with 15 μL denaturing Laemmli buffer for 10 min at 95 °C, and 10 μg of the total proteins was used as input. Immunoprecipitated and input protein were processed by immunoblotting, and ODs were corrected to Actin or IgG as the loading control.

### 2.6. Quantitative PCR 

Total RNA was extracted and purified from both non-HHS and HHS-treated ARPE-19 cells using the RNeasy mini plus kit (Qiagen: Hilden, Germany, 74034), and 1 μg RNA was retrotranscribed to cDNA using iScript cDNA synthesis kit (Bio-Rad Laboratories, 1708891). The expressions of the transcripts were assayed with using iQ SYBR green supermix (Bio-Rad Laboratories, 1708880) in a CFX real-time thermal cycler (Bio-Rad Laboratories, Hercules, CA, USA). The PrimePCR used were HSP70 (AssayID: qHsaCED0037313); BECN1 (AssayID: qHsaCID0016032); ATG7 (AssayID: qHsaCID0012170); ATG5 (AssayID: qHsaCID0018096); MAP1LC3B/LC3B (AssayID: qHsaCED0038576); SQSTM1 (AssayID: qHsaCED0045925); RAB7 (AssayID: qHsaCID0008324); and LAMP2 (AssayID: qHsaCID0020863). The mRNA levels of the target genes were corrected to the mean of the housekeeping genes: RPL13 (AssayID: qHsaCED0045063) and TBP (AssayID: qHsaCID0007122). The relative transcript expression levels for HHS samples were normalized to non-HHS applying the ΔΔCT method. All paired oligos for PrimePCR are from Bio-Rad Laboratories.

### 2.7. Immunofluorescence

Cells were cultured on glass coverslips and treated with HHS. Cells were rinsed with phosphate-buffered saline (PBS; ThermoFisher Scientific, 10010023) and fixed with formaldehyde (FA; Solveco: Rosersberg, Sweden, 50-00-0) in PBS for 10 min at RT, followed by permeabilization with PBS supplemented with 0.5% Triton X-100 (Sigma-Aldrich, No.9002-93-1) for 15 min at RT. Permeabilized cells were incubated in a blocking solution of 0.1% PBS-T supplemented with 10% normal goat serum (ThermoFisher Scientific, 10000C) for 1 h at RT. Then, the cells were incubated ON at 4 °C with primary antibodies diluted in a blocking solution: HSP70 (1:200, mouse polyclonal; Santa Cruz Biotechnology, SPA-810); anti-SQSTM1 (1:200, rabbit monoclonal; Novus Biologicals, NBP1-48320); anti-LC3B (1:200, rabbit monoclonal; Cell Signaling Technology, 3868); and anti-HSF1 (1:150, rabbit monoclonal; Cell Signaling Technology, 12972D). Incubation with secondary antibodies diluted in a blocking solution was performed for 1 h at RT: Anti-mouse-Alexa 647 (1:500; ThermoFisher Scientific, A32787), Anti-rabbit-Alexa 488 (1:500; ThermoFisher Scientific, A-11034), Anti-rabbit-Alexa 546 (1:500; ThermoFisher Scientific, A-11010), Alexa Fluor 488-Phalloidin (1:500; ThermoFisher Scientific, A12379), and Hoechst 33258 (1:10,000; Sigma-Aldrich, 23491-45-4). Finally, immunofluorescence (IF) was post-fixed for 5 min in FA at RT, and coverslips were mounted with fluorescence mounting medium (Dako, CS70330-2). Images were acquire using a Zeiss fluorescence microscope with the AxioVision software version 4.8 (Zeiss, Gottingen, Germany) by applying fixed camera exposures for equative signal across the same channel.

### 2.8. Autophagic Flux Detection 

The Premo™ Autophagy Tandem Sensor RFP-GFP-LC3B Kit (ThermoFisher Scientific, P36239) was used to evaluate the autophagosome. Tandem sensor RFP-GFP-LC3B was used as described in the manufacturer’s instructions. Briefly, cells were cultured on glass coverslips to ~70% confluency and exposed to 40 BacMam particles per cell ON, followed by Baf treatment as described. Then, the cells were rinsed and fixed with FA for 10 min at RT. The nuclei were stained with Hoescht at 1:10,000 (Sigma-Aldrich, 23491-45-4) for 5 min and post-fixed before mounting with fluorescent mounting medium (Dako, CS70330-2).

The pH independent LysoTracker Red DND-99 (ThermoFisher Scientific, L7528) was used to evaluate the lysosomes in the autophagy flux. Cells cultured on glass coverslips and treated with Baf and HHS were exposed to 75 nM LysoTracker during the final 2 h of treatment. The fixed cells were co-stained with SQSTM1 and Hoescht following the IF protocol.

To enhance the visualization of the puncta, autophagy flux images were acquired with a Zeiss fluorescence microscope and corrected to maximum projection using the AxioVision software version 4.8 (Zeiss, Gottingen, Germany); co-localization measurements were performed after subtracting the backgrounds and applying maximum fluorescence intensity threshold. Sixty cells displaying triple staining from three independent experiments were analyzed by counting and measuring the area of puncta.

### 2.9. Statistical Analysis 

The statistical analyses were calculated using Prism software version 8 (GraphPad Inc., San Diego, CA, USA). The data were subjected to one-way analysis of variance (ANOVA), corrected by Sidak or Bonferroni post hoc tests for multiple comparisons, as described in figure legends. The data were presented as mean ± standard error of mean (SEM), and values of *p* < 0.05 were considered statistically significant. 

## 3. Results

### 3.1. Hormetic Heat Shock Induces a Long-Lasting Heat Shock Response in ARPE-19 Cells

**Figure 1:**
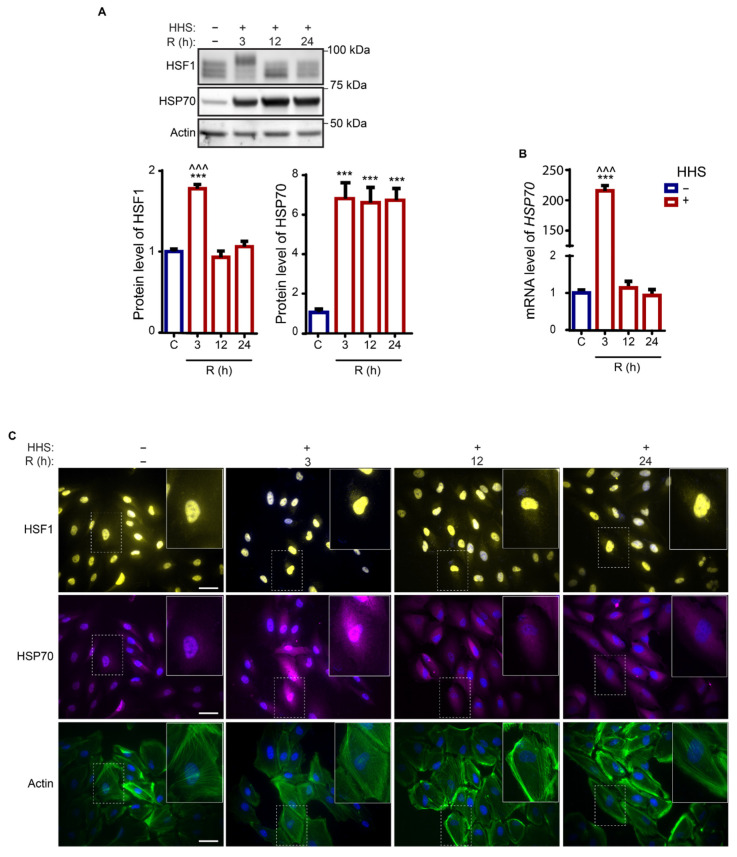
Hormetic heat shock induces heat shock response in ARPE-19 cells. ARPE-19 cells were treated without HHS (−) or with 30 min HHS (+) at 42 °C, followed by recovery at 37 °C. (**A**) Cell lysates were subjected to immunoblot analysis with HSF1 and HSP70, and Actin. The quantitative analysis of protein level was corrected to Actin as a loading control. (**B**) mRNA expression of the *HSP70* gene evaluated with qPCR. mRNA levels for the HHS samples were normalized to non-HHS applying the ΔΔCT method. (**C**) Immunofluorescence analysis was illustrated for HSF1 (yellow), HSP70 (magenta), and Actin (green). Cells’ nuclei were counterstained with Hoechst (blue). C stands for control (non-HHS). R shows the post-HHS time of recovery at 37 °C. Data are represented as mean ± SEM (*n* = 3 for (**A**) and *n*= 6 for (**B**)) and statistical analysis was performed using one-way ANOVA, followed by Bonferroni‘s multiple comparisons test (*** *p* < 0.001 vs. C, ^^^ *p* < 0.001 vs. 12 and 24). Scale bar 20 µm.

To investigate the mechanisms engaged in the HHS responses in ARPE-19 cells, we first examined the time recovery behavior of the canonical heat shock responders, HSF1 and HSP70, to a 30 min 42 °C heat shock (Figure 1). The immediate response of HHS after 3 h recovery demonstrated a significant increase in HSF1 protein expression levels (*p* < 0.001 vs. non-HHS; Figure 1A). The exposure to HHS resulted in the observed electrophoretic upshift of the HSF1 protein immunosignal due to post-translational modifications, correlated to the activation of the transcription factor [39,40]. The HSR of this treatment demonstrated 200-fold induction of the *HSP70* gene transcript (*p* < 0.001 vs. non-HHS; Figure 1B) and an observable nuclear translocation of HSF1 at 3 h post-HHS (Figure 1C). On later times of recovery, HSF1 expression was reverted to the non-HHS level (Figure 1A). In agreement, immunofluorescence imaging demonstrated diminished localization of the HSF1 protein in the cytosol and elevated accumulation in the nucleus at the 3 h time point and redistribution to cytosolic and nuclear localization at later times, suggesting deactivation of the transcription factor (Figure 1C). This observation is in agreement with previous studies on different cell lines [40,41]. However, protein analysis demonstrated sustained upregulation of HSP70 expression in the studied recovery time frame (*p* < 0.001 vs. non-HHS; Figure 1A). The sub-cellular localization of HSP70 was largely cytosolic and remained unaltered by HHS and during recovery (Figure 1C). Cell morphology was monitored using fluorescent-labelled phalloidin to visualize polymerized Actin filaments. No discernable changes in the morphology or cytoskeleton organization of the ARPE-19 cells due to HHS were observed (Figure 1C). Collectively, hormetic heat did not alter the morphology of RPE cells, and the short-term induction of HSF1 was followed by the long-lasting expression of HSP70.

### 3.2. Hormetic Heat Shock Increase Autophagy Gene Expression by HSF1 Transcriptional Activation

**Figure 2:**
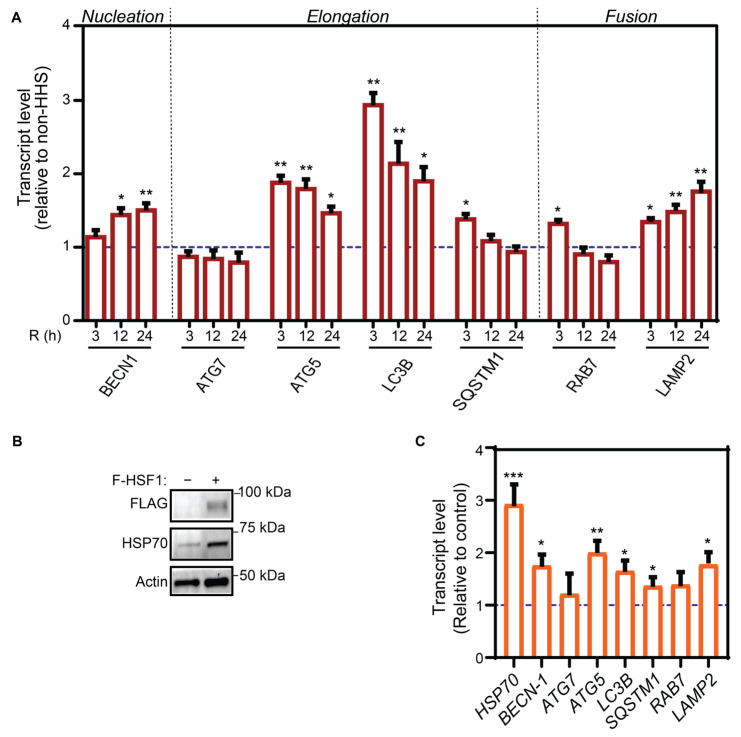
Hormetic heat response regulates the induction of autophagy-associated genes. (**A**) ARPE-19 cells were treated without HHS (–) or with 30 min HHS (+) at 42 °C, followed by recovery at 37 °C. The expression level of transcripts involved in various stages of autophagy was analyzed by qPCR. (**B**,**C**) ARPE-19 cells were transfected with the FLAG-HSF1 expression construct at 37 °C. (**B**) Immunoblot analysis of FLAG, HSP70, and Actin for cells overexpressing HSF1 were illustrated with representative images. (**C**) Transcript levels of genes involved in various steps of the autophagy process in ARPE-19 cells under HSF1 gain-of-function were evaluated with qPCR. The dashed line (---) stands for non-HHS (**A**) or mock-transfected controls (**C**). R represents the post-HHS time of recovery at 37 °C. Data are reported as mean ± SEM (*n* = 6) and statistical analysis was achieved using one-way ANOVA, followed by Sidak test (* *p* < 0.05, ** *p* < 0.01, *** *p* < 0.001).

Previous studies reveal that HHS may prevent age-related detrimental protein aggregation [42,43]. Therefore, to analyze the effects of HHS on autophagy activity in ARPE-19 cells, a set of autophagy-associated genes involved in the different phases of the autophagic flux, including nucleation (*BECN1*), elongation (*ATG7*, *ATG5*, *LC3B*, and *SQSTM1*), and fusion (*RAB7*, *LAMP2*) were selected for analysis by qPCR (Figure 2A). Gene expression data revealed two different profiles: early and late responding genes. The early response genes, including *ATG5*, *LC3B*, *SQSTM1*, and *RAB7*, displayed a rapid and statistically significant upregulation (*p* < 0.01, *ATG5* and *LC3B* and *p* < 0.05, *SQSTM1* and *RAB7* vs. non-HHS), showing the maximum mRNA level at the earliest (3 h) recovery time point and gradually decreasing after longer recovery times. Instead, the late response genes *BECN1* and *LAMP2* demonstrated significant progressive increase (from *p* < 0.05 to *p* < 0.01 vs. non-HHS) of their mRNA levels up to the 24 h recovery time point. The transcript level of *ATG7* did not vary in response to HHS.

The benefits of hormesis have been shown to be mediated by the activation of HSR regulated by HSF1 [44]. We further explored whether the gain-of-function of HSF1 can transcriptionally upregulate the expression of autophagy-associated genes in RPE cells. In this regard, ARPE-19 cells were transfected with a FLAG-tagged HSF1 expression construct and demonstrated an observable increase in the expression of HSP70 (Figure 2B) as well as a statistically significant increase in the *HSP70* gene expression (Figure 2C), validating the activation of HSF1. Moreover, the expression of genes including *BECN1*, *ATG5*, *LC3B*, *SQSTM1,* and *LAMP2* was significantly higher in cells overexpressing HSF1 than in cells under basal (non-HHS) conditions (Figure 2C). These data demonstrated that increased expression and transcriptional activity leads to upregulated expressions of autophagy genes, including those involved in autophagosome formation and lysosome degradation. Altogether, the transient HHS-induced activation of autophagy genes is caused by a time-dependent activation of HSF1.

### 3.3. Hormetic Heat Shock Increases Autophagic Flux

**Figure 3:**
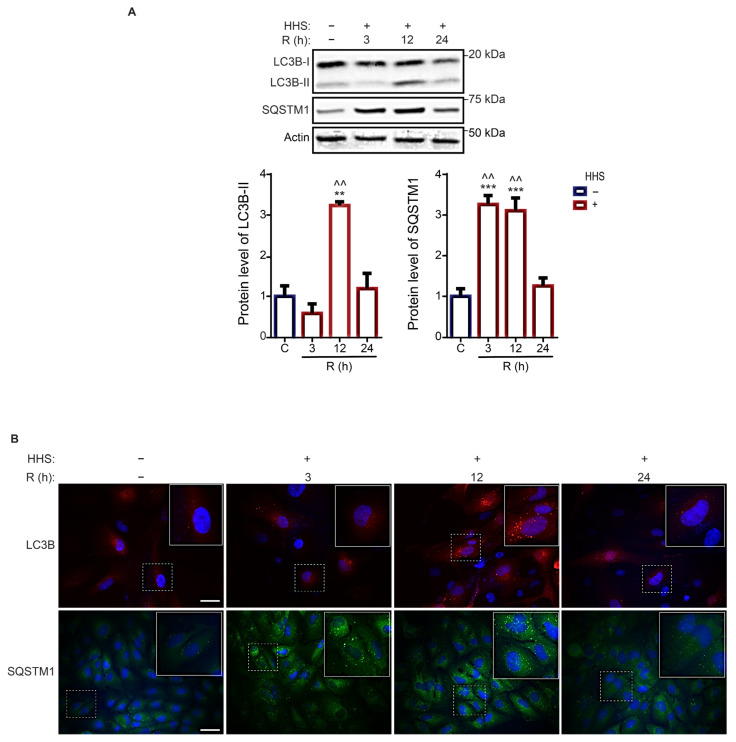
Hormetic heat shock increases expression of autophagosome-related proteins. ARPE-19 cells were treated without HHS (–) or with 30 min HHS (+) at 42 °C, followed by recovery at 37 °C. (**A**) Immunoblot quantitative analysis of the LC3B-II and SQSTM1 levels was corrected to Actin as a loading control. (**B**) Immunofluorescence analysis was shown for the puncta of LC3B (red) and SQSTM1 (green). Cells’ nuclei counterstained blue with Hoechst. C stands for control (non-HHS). R shows the post-HHS time of recovery at 37 °C. Data are represented as mean ± SEM (*n* = 3). Statistical analysis was used by one-way ANOVA followed by Bonferroni‘s multiple comparisons (** *p* < 0.01 and *** *p* < 0.001 vs. C, ^^ *p* < 0.01 vs. 24). Scale bar = 20 µm.

As autophagy is a complex pathway with contributions from a myriad of proteins, the expressions of LC3B and SQSTM1, two canonical autophagy proteins vital for autophagosome formation in the elongation step and cargo recognition, were further monitored in ARPE-19 cells exposed to HHS. The protein level of LC3B-II demonstrated a significant increase at 12 h recovery time point compared with non-HHS (*p* < 0.01), albeit normalized at 24 h recovery (Figure 3A). Subcellular distribution of both LC3B and SQSTM1 into a punctate pattern is considered to validate the formation of autophagosomes. In agreement with the immunoblot analysis, IF illustrated an increase in LC3B puncta at 12 h with a decrease at 24 h post-HHS (Figure 3B). In addition, HHS significantly increased the SQSTM1 protein level (*p* < 0.001 vs. non-HHS), followed by a normalization to non-HHS levels at 24 h recovery (Figure 3A). A concomitant SQSTM1 puncta pattern was observed by IF (Figure 3B).

**Figure 4:**
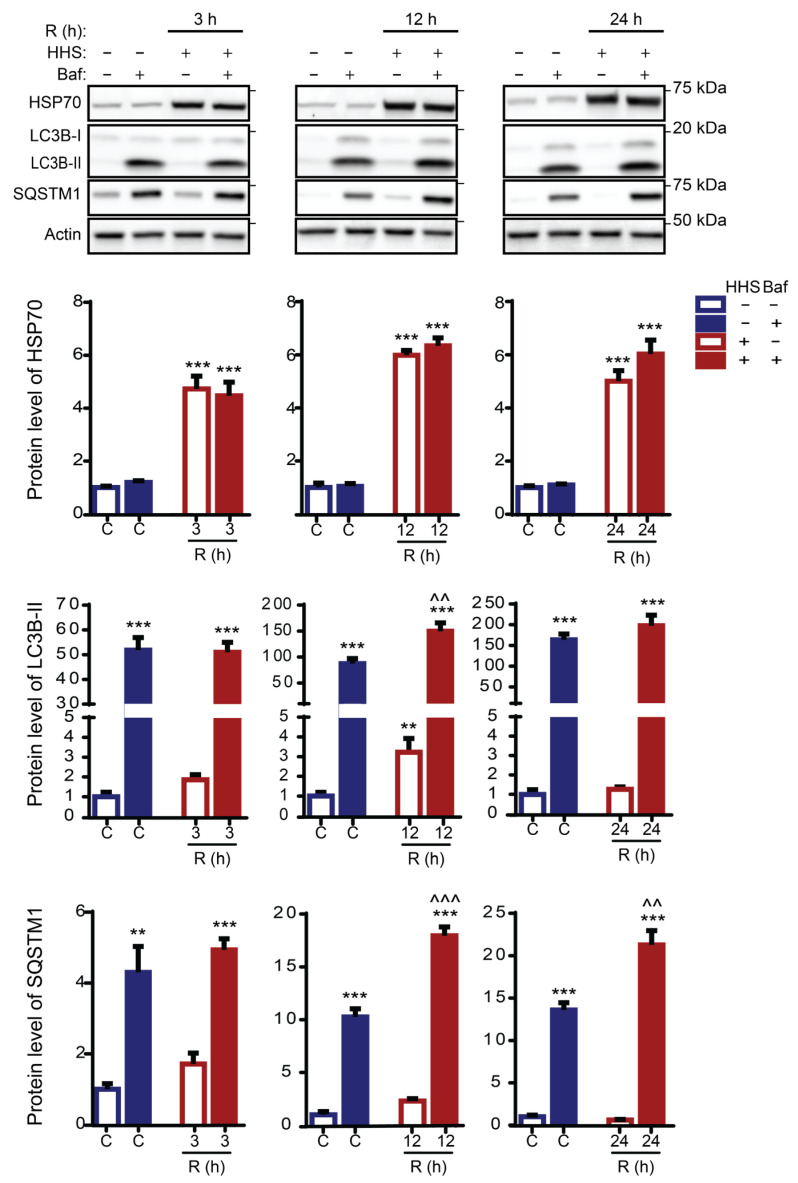
Hormetic heat shock induces autophagy. ARPE-19 cells were exposed initially for 6 h to 50 nM Bafilomycin (Baf); then, cells were treated without HHS (–) or with 30 min HHS (+) at 42 °C, followed by recovery at 37 °C in the presence of Baf. Immunoblot analysis of HSP70, LC3B-II, and SQSTM1 are illustrated with representative images. C stands for Non-HHS/Non-Baf and Non-HHS/Baf treated cells. R shows the post-HHS time of recovery at 37 °C. The quantitative analysis was corrected to Actin as a loading control and normalized to Non-HHS/Non-Baf as the control. Data are represented as mean ± SEM (*n* = 3), followed by one-way ANOVA by Sidak test (** *p* < 0.01 and *** *p* < 0.001 vs. C, ^^ *p* < 0.01, and ^^^ *p* < 0.001 vs. Baf).

The observed increase in LC3B-II protein level and LC3B puncta distribution could result from enhanced autophagy induction or from inhibition of the autophagic flux [45]. To distinguish between these possibilities, Bafilomycin A1 was used to evaluate the impact of HHS on ARPE-19 cells. Bafilomycin A1 is a V-ATPase inhibitor that inactivates autophagosomal turnover and prevents acidification of the lysosome. Immunoblot analysis demonstrated that Bafilomycin A1 did not affect the amount of HSP70 in ARPE-19 cells (Figure 4). Instead, HHS induced a significant and similar expression of HSP70 at all recovery times assessed (*p* < 0.001 vs. non-HHS), regardless of whether Baf was present or not. These data validate that HSP70 overexpression is due to hormetic HSR and independent of Baf treatment (non-significant vs. HHS/Baf; Figure 4).

Inhibition of lysosomal activity by Baf led to a significant accumulation of both LC3B-II and SQSTM1 (*p* < 0.001 and *p* < 0.01 vs. non-HHS/non-Baf). In the presence of Baf, the amount of LC3B-II demonstrated a significant HHS-induced increase at 12 h recovery (*p* < 0.01 vs. non-HHS/Baf), in line with a significant HHS-induced elevation at 12 h without Baf (*p* < 0.01 vs. non-HHS/non-Baf). However, no significant additional HHS-induced accumulation of LC3B-II could be observed at 24 h. The HHS-induced increase in SQSTM1 expression was significant at 12 and 24 h recovery (*p* < 0.001 and *p* < 0.01 vs. non-HHS/Baf). The HHS-induced additional accumulation of LC3B-II and SQSTM1 proteins in the presence of Bafilomycin (compared with Baf alone) demonstrates that HHS enhances the autophagic flux in RPE cells.

### 3.4. HHS-Induced Increase in Autophagic Flux Does Not Depend on HSP70

**Figure 5:**
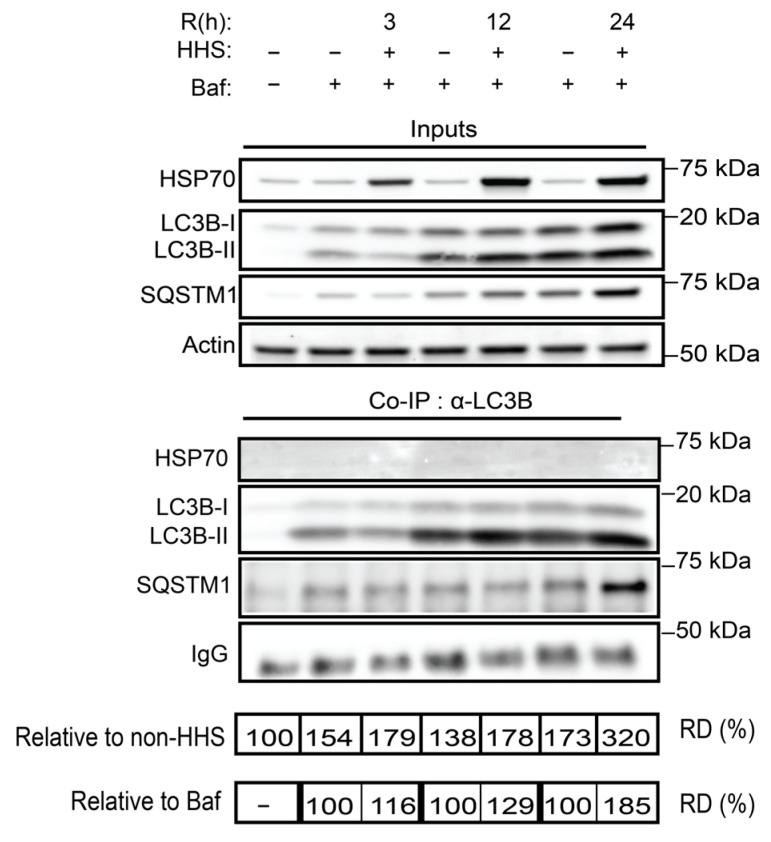
LC3B associated interactome in the presence of HHS. ARPE-19 cells were exposed initially for 6 h to 50 nM Bafilomycin (Baf); then, cells were treated without HHS (–) or with 30 min HHS (+) at 42 °C, followed by recovery at 37 °C in the presence of Baf. Immunoprecipitation of the LC3B was performed with LC3B antibody to identify interactions between LC3B/HPS70 and LC3B/SQSTM1 complex. The protein complex formation was normalized to the IgG as loading control. R shows the post-HHS time of recovery at 37 °C. The complex interaction status was determined by relative densitometry (RD) in percentage compared to either Non-HHS/Non-Baf or Non-HHS/Baf. The immunoprecipitation was performed from a pool of six independent experiments.

LC3B and SQSTM1 play significant roles in autophagic flux, and previous studies have described the interactions between LC3B and SQSTM1 as well as between HSP70 and SQSTM1 [45,46]. However, a putative interaction between HSP70 and LC3B as a result of HHS has not been characterized. To elaborate on whether hormetic HSR mediates a putative LC3B/HSP70 interaction or alters the LC3B/SQSTM1 complex, a possible role for HSP70 in the regulation of autophagy was assessed by a co-immunoprecipitation analysis (Figure 5). Protein complexes isolated by immunoprecipitation of LC3B did not suggest an interaction with HSP70. Expectedly, an analysis of the co-immunoprecipitated protein complex demonstrated a clear interaction between LC3B and SQSTM1. Relative densitometry (RD) of complexed SQSTM1 demonstrated that exposure to HHS increased its interaction with LC3B, particularly when compared with non-HHS/Baf. Together, these data indicated that hormetic heat enhanced the autophagic flux through increased interaction between LCB3 and SQSTM1 but in an HSP70-independent manner.

### 3.5. Hormetic Heat Shock Increases the Size of Autophagosomes and the Presentation of SQSTM1-Associated Cargo to Lysosomes

**Figure 6:**
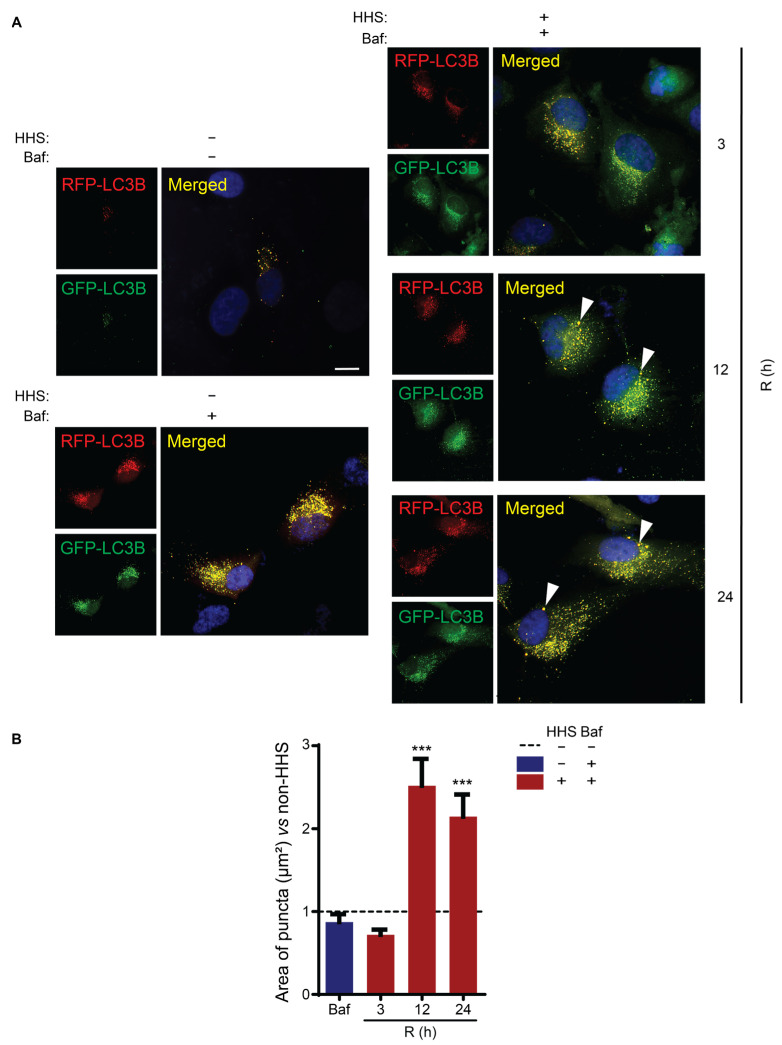
Hormetic heat shock increases size of autophagosome. ARPE-19 cells were transfected with RFP-GFP- LC3B construct for 16 h; then, were exposed initially for 6 h to 50 nM Bafilomycin (Baf). Later, the cells were treated without HHS (–) or with 30 min HHS (+) at 42 °C, followed by recovery at 37 °C in the presence of Baf. Non-HHS/Non-Baf were used as a control. (**A**) Immunofluorescence analysis was illustrated for autophagosome puncta (yellow) and autolysosome (red). Nuclei was counterstained with Hoechst (blue), and the arrowhead points to autophagosomes. (**B**) The area of yellow puncta (scored autophagosome’s size) was analyzed per sample and quantitative analysis was normalized to Non-HHS/Non-Baf, represented as a dashed line (---). R shows the post-HHS time of recovery at 37 °C. Statistical analyses are shown as mean ± SEM (*n*= 60) followed by one-way ANOVA by Sidak test (*** *p* < 0.001 vs. C). Scale bar = 20 µm.

The interaction of LC3B with SQSTM1 occurs mainly during the elongation phase of autophagy and is associated with cargo presentation into the autophagosome [8]. Therefore, to investigate whether the increased interaction of the LC3B/SQSTM1 complex in response to HHS would affect the elongation step and lead to an increase in the size of autophagosomes, the effect of hormetic heat shock on the LC3 puncta was evaluated using the tandem RFP-GFP-LC3B construct (Figure 6). LC3B tagged with both pH-dependent GFP and pH-independent RFP enabled differentiation of autophagosomes from autolysosomes; the higher sensitivity of GFP to the acidic environment inside lysosomes leads to quenching of the GFP signal upon fusion of autophagosome to lysosome during autolysosome formation. Thus, autophagosomes are visualized as yellow puncta, while autolysosomes are visualized as red puncta. Notably, the use of Baf prevents acidification of lysosomes and therefore quenching of GFP, resulting in yellow LC3B puncta [47]. Fluorescence microscopy illustrated that exposure of ARPE-19 cells to Bafilomycin A1 had no observable effects on the size of LC3B-positive structures (Figure 6A, non-HHS/Baf vs. non-HHS/non-Baf). Instead, exposing the cells to HHS in the presence of Baf appeared to increase the size of LC3B puncta at 12 and 24 h post-HHS (Figure 6A, HHS/Baf). To further elaborate on autophagosome enlargement, the area of the LC3B-positive structures was measured (Figure 6B). The quantitative analysis demonstrated that HHS/Baf treatment significantly increased the area of individual LC3 puncta at 12 and 24 h (both *p* < 0.001 vs. non-HHS/non-Baf), while Baf treatment alone did not affect the size of LC3B-positive structures.

**Figure 7:**
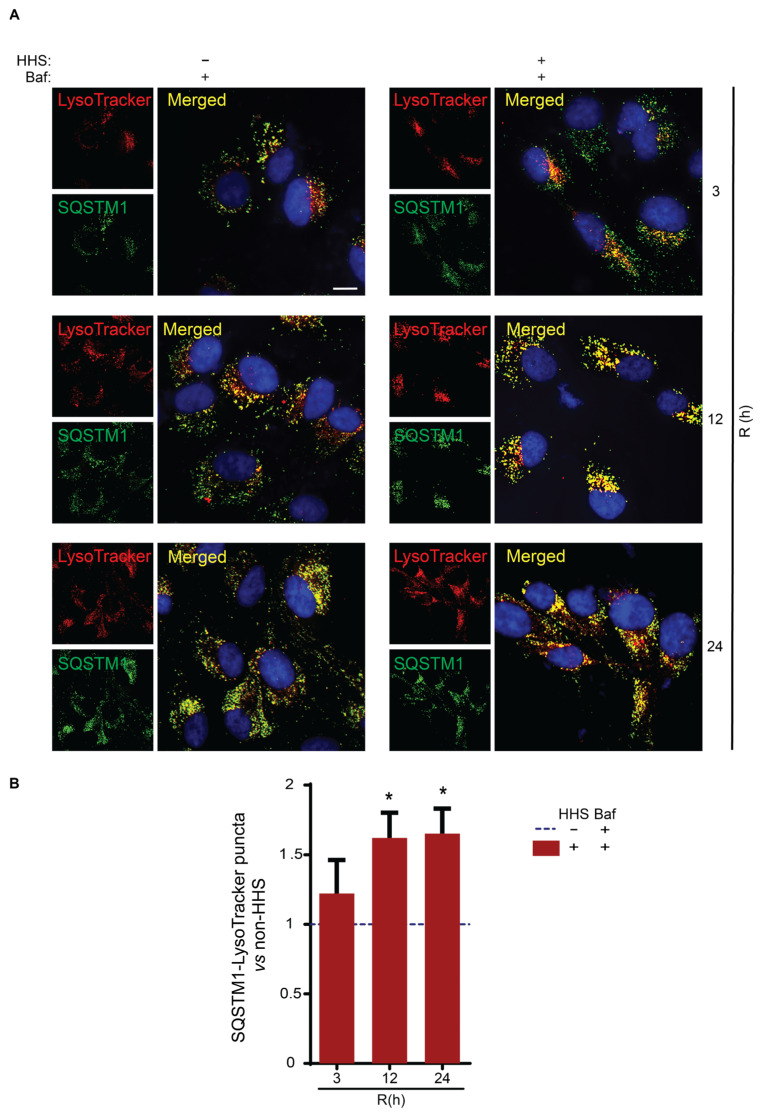
Hormetic heat shock enhances SQSTM1-associated cargo presentation to lysosome. ARPE-19 cells were exposed initially for 6 h to 50 nM Bafilomycin (Baf); then, cells were treated without HHS (–) or with 30 min HHS (+) at 42 °C, followed by recovery at 37 °C in the presence of Baf. Cells were exposed to LysoTracker during the final 2 h treatment. (**A**) Colocalization of SQSTM1 (green) with LysoTracker (red) was examined by IF. Cells’ nuclei were counterstained with Hoechst (blue). (**B**) The number of yellow puncta (scored as colocalized SQSTM1-LysoTracker) was counted per sample, and quantitative analysis was normalized to Non-HHS/Baf, represented as dashed line (---). Data are shown as mean ± SEM (*n* = 60). Statistical analysis was performed using one-way ANOVA, followed by Sidak test (* *p* < 0.05). Scale bar = 20 µm.

The observed increase in the size of autophagosomes as a consequence of hormetic heat suggested improved sequestration of cargo during autophagy. Since SQSTM1 is a known carrier responsible for the presentation of multiple substrates to the autophagosome for subsequent degradation in the autolysosome, we assessed whether hormetic heat improves the presentation of SQSTM1 to the lysosome. Therefore, a pH-independent LysoTracker, which fluorescently labels all lysosomal compartments, was used to determine the SQSTM1 puncta localization (Figure 7). IF displayed a discernible increase in the number of yellow puncta, suggesting a population considered as SQSTM1 localized to lysosomal compartments at 12 and 24 h post-HHS in cells exposed to HHS/Baf (Figure 7A). Accordingly, the quantification analysis of lysosomal-SQSTM1 puncta confirmed a significant increase of ~50% in the number of puncta in HHS-treated cells (*p* < 0.05 vs. non-HHS/Baf; Figure 7B) both at 12 and 24 h. Collectively, these findings indicated that HHS increased the size of autophagosomes and enhanced the presentation of SQSTM1-associated cargo for the lysosome.

## 4. Discussion

In this study, we demonstrated novel evidence that hormetic heat shock activates autophagy in human RPE cells. HHS induces a short-term activation of HSF1 followed by a long-term expression of HSP70, a canonical heat shock responder. Furthermore, our study highlights that the activation of HSF1 by HHS mediates the expression of several autophagy genes, resulting in enlarged autophagosomes and an enhancement in the SQSTM1-mediated cargo presentation to the lysosomes, which does not depend on HSP70 in line with our earlier report [48]. Based on these findings, our results indicate that hormetic heat shock increases the induction of autophagic flux in RPE cells.

Several studies in retinal disease have been directed to apply a hormetic increase in temperature to activate the HSR yet prevent cellular damage [23,24,25]. Accordingly, the induction of HSPs in RPE cells after transpupillary laser irradiation [24,25,26,27,28,29] could lead to therapeutical effects [24,25,30,31,32]. However, hormetic heat shock can affect and modulate a multitude of mechanisms, e.g., a corresponding heat shock led to an upregulation of nearly 1500 genes and downregulation of approximately 8000 genes in mouse embryonic fibroblasts during the first hour of heat shock [49]. Thus, the molecular mechanisms underlying the beneficial effects of HHS response may remain largely uncharacterized yet. 

The conventional heat shock response is generated by a conserved signaling pathway that regulates the activity of HSFs, such as HSF1, in which activation leads to the expression of heat shock proteins acting as molecular chaperones [50,51,52]. Among the three major protection mechanisms responsible for maintaining the cellular proteostasis (the heat shock proteins, the UPS, and autophagy), the HSPs are the only class of proteins that has the ability to refold and repair unfolded or misfolded proteins. Upon activation of HSFs, phosphorylation and other post-translational modifications followed by the formation of homotrimers of HSF1 are responsible for the heat shock response, leading to the increased synthesis of HSPs [50,51]. This was confirmed in our study on ARPE-19 cells, as an early response of HSF1 at 3 h post-HHS resulted in a significant increase in *HSP70* mRNA and HSF1 protein levels but both had returned to their non-HHS levels within 12 h, which is probably due to the HSF1 autoregulation by HSPs [50]. On the contrary, the transcriptional activation of *HSP70* promoted a significant and long-term expression of HSP70 in RPE cells, suggesting a long-lasting HHS impact on the translation of HSP70 and therefore elevated capacity for maintenance and repair of proteins. Although our data shows no recovery of HSP70 towards the basal level, the HSP70 protein level had started to decrease at 48 h post-HHS (data not shown).

Recent data suggest that the role of HHS is not limited to the expression of HSP70 and other HSPs. Heat treatment has been shown to increase autophagy induction in *C. elegans*, human malignant glioma cells, and rat hepatocytes [53,54,55]. An efficient autophagic flux in RPE cells is required to allow for proper nutrient recycling and removal of protein aggregates and dysfunctional organelles [2], and impaired autophagy has been associated with multiple neurodegenerative diseases [56], including AMD [33,34,35]. Since RPE cells obtained from AMD donors show lower autophagic flux compared with RPE cells from healthy controls [33,34,35], we considered whether moderate heating might activate autophagy in human RPE cells, thereby possibly providing the means to a heat-controlled waste clearance and improved proteostasis in RPE cells. We found that hormetic heat shock and the overexpression of HSF1 significantly upregulates several key autophagy-associated genes in ARPE-19 cells while none of the genes examined in this study was downregulated. In line with a study on *C. elegans*, our findings clarify an HHS-mediated HSF-transcriptional activation in ARPE-19 cells that upregulate a set of autophagy-associated genes [53]. In addition, we found that the HHS-induced upregulation of the autophagy-associated genes featured two different profiles, early and late responding genes. Among the early response genes, there are genes involved in phagophore expansion and autophagosome formation (*ATG5*, *LC3B,* and *SQSTM1*) as well as in the fusion step between autophagosome and lysosome (*RAB7*), while the two late responding genes found in the chosen set of seven genes are involved in the early phase of autophagy induction, the nucleation phase of the isolation membrane (*BECN1*), and in the late phase of autophagy, the fusion of autophagosome and lysosome (*LAMP2*).

Although the canonical heat shock response inducing the elevated production of heat shock proteins has been studied extensively, the observation that autophagy might be initiated by moderate heat shocks has emerged relatively recently, and much of the present data on heat induction of autophagy-related genes comes from studies in *C. elegans* [53]. Many genes contain HSEs in their promoter region, which identifies them as HSF-regulated target genes. Since HSFs are rapidly activated by heat shock, HSF1 is a potential initiator candidate for the early responding genes. Previous to our study, among the mammalian genes studied, only the *SQSTM1* gene has been identified to contain an HSE in its promoter region and be HSF1-upregulated (for a review of HSF1 target genes, see [57]). Here, we identified five autophagy-associated genes, including *SQSTM1*, *BECN1*, *ATG5*, *LC3B,* and *LAMP2* to be upregulated in response to overexpression of HSF1, suggestive of HSF1-mediated transcriptional activation of these genes in human retinal cells. A study in *C. elegans* has identified over 40 autophagy-related genes contain at least one putative HSE, including *atg-7*, *bec-1*, *lgg-2*, *sqst-1*, and *vps-34* [53]. However, it has been suggested that binding of HSF1 to an HSE does not show induction in all genes [58]. This could imply that at least some of the HHS-induced autophagy-related genes might not be under direct transcriptional control of HSF1, albeit HSF1-associated alternative epigenetic regulation may be involved. Thus, much research is needed to understand the activation pathways and the biological role of the heat-induced early and late responses of autophagy-associated gene, as identified by us following a time-dependent activation of active HSF1 in human RPE cells.

Consistent with the early induction of the *SQSTM1* gene by HHS, we found a significant increase in the SQSTM1 protein level at 3 and 12 h after HHS. In the cytosol, SQSTM1 can bind to polyubiquitin chain-tagged proteins destined for destruction and therefore spontaneously create SQSTM1 droplets that have gel-like properties. The SQSTM1 droplets can exchange their components with the environment and recruit core ATG proteins necessary for autophagosome formation [59,60,61]. We hypothesized that the observed HHS-induced increase in SQSTM1 and in other core proteins involved in phagophore nucleation and elongation (BECN1, ATG5, and LC3B-II) would lead to the formation of larger SQSTM1 droplets and consequently to larger autophagosomes. Indeed, this was confirmed by our immunofluorescence analysis on autophagosome puncta, leading us to conclude that hormetic heat shock may result in enhanced condensation of SQSTM1 cargo, manifested as larger autophagosomes, and thus potentially lead to more effective waste clearance by selective autophagy in human RPE cells.

The interaction between LC3B and SQSTM1 in selective autophagy as well as between HSP70 and SQSTM1 in chaperone-assisted selective autophagy has been described [45,46]. Using HSP70 overexpressing cell lines and inhibiting HSF-1 expression with siRNA, Dokladny et al. showed that high levels of HSP70 lead to low LC3-I and LC3-II levels in human cancer cells [62]. This interaction was shown to be independent of HSF-1, suggesting that HSP70 rather than HSF-1 may regulate the LC3 levels. Therefore, we investigated using immunoprecipitation whether HSP70 and LC3B could form protein complexes as response to HHS-mediated autophagy. An interaction between HSP70 and LC3B could not be identified, indicating that the observed enlarged autophagosomes identified in ARPE-19 cells are SQSTM1-mediated yet HSP70-independent. This new evidence is in agreement also with our previous findings, demonstrating that HSP70 is selectively involved in proteasome-mediated clearance of protein aggregates rather than macroautophagy [48].

In yeast, autophagic activity is regulated by modulating both the number and the size of autophagosomes [63]. Based on a study using nitrogen starvation or rapamycin treatment, leading to elevated ATG8 level and enlarged autophagosomes, the researchers of Klionsky lab concluded that ATG8, the yeast homolog of the human LC3B protein, is a key protein in controlling the growth of phagophores and that enhanced amounts of ATG8 are necessary for larger autophagosomes [64]. Our result that HHS substantially raises the level of LC3B-II and enhances the size of autophagosomes is in line with the yeast data, suggesting a parallel mechanism in mammalian cells during HHS response. In addition to larger autophagosomes, we also observed an increase in the number of LC3B and SQSTM1 puncta in response to HHS. We also demonstrated that the SQSTM1 puncta colocalized with lysosomes under bafilomycin treatment, a condition that blocks the lysosomal degradation of autophagic cargos and allows for colocalization examinations. The SQSTM1-lysosome colocalization suggests that the observed puncta most likely represent autophagosomes instead of being random focal accumulations of SQSTM1.

The observed HHS-induced increase in the level of the key autophagy proteins LC3B-II and SQSTM1 may be interpreted that heat either can boost the production of autophagy-associated proteins and thus accelerate autophagic flux, or that heat inhibits autophagy and therefore causes the accumulation of LC3B-II and SQSTM1. The first clue that HHS improved autophagic flux in our experiments on ARPE-19 cells comes from the observation that the heat-induced elevation of both LC3B-II and SQSTM1 proteins was almost completely removed at 24 h post-HHS, suggesting that autophagy is active after heat shock. This conclusion is supported by the result that, under bafilomycin treatment, which inhibits lysosomal cargo degradation and thus strongly increases the accumulation of LC3B-II and SQSTM1, HHS was able to elevate still the levels of these proteins. Furthermore, the observed HHS-induced increase in the expression of six out of the seven key autophagy-associated genes is in line with our interpretation that HHS activates autophagy in human ARPE-19 cells.

A lack of proper RPE cell lines hinders the development of in vitro AMD models. The human RPE cell line ARPE-19 is widely used to study molecular mechanisms, but ARPE-19 cells tend to lose the original phenotype of the RPE. hES and hiPS cells have been differentiated into functional RPE cells and present alternatives for in vitro AMD models, although such cultures require time consuming protocols. Currently, our study has been limited to ARPE-19 cells, and further investigation in other RPE cells, including animal models, would contribute to extending our findings in ophthalmic diseases.

## 5. Conclusions

Our study provides evidence that hormetic heat shock boosts the expression of several fundamental autophagy-associated genes through activation of HSF1, transiently raises the level of SQSTM1 and LC3B-II proteins crucial for autophagic cargo building and autophagosome formation, and activates autophagy in human retinal pigment epithelial cells (see graphical abstract). Our results suggest that HHS might provide improved cellular waste clearance and proteostasis, which could be beneficial under cytotoxic conditions and in age-related neurodegenerative diseases. As HHS transiently activates both the HSP70 expression and selective autophagy in human RPE cells, transpupillary near-IR laser heating of the RPE might provide a means to improving the cytoprotective capacity of the RPE cells at the fundus of the eye. However, the therapeutic temperature window for subthreshold heat shock is narrow and requires accurate temperature control of the RPE during heating [26]. This may be achieved by the retinal temperature determination method developed in the research group of Koskelainen [65,66,67], thereby counteracting the progress of age-related macular degeneration. 

## References

[1] Kuusisto E., Salminen A., Alafuzoff I. (2002). Early Accumulation of P62 in Neurofibrillary Tangles in Alzheimer’s Disease: Possible Role in Tangle Formation. Neuropathol. Appl. Neurobiol..

[2] Kaarniranta K., Salminen A., Haapasalo A., Soininen H., Hiltunen M. (2011). Age-Related Macular Degeneration (AMD): Alzheimer’s Disease in the Eye?. J. Alzheimer’s Dis..

[3] Braak H., Thal D.R., Del Tredici K. (2011). Nerve Cells Immunoreactive for P62 in Select Hypothalamic and Brainstem Nuclei of Controls and Parkinson’s Disease Cases. J. Neural Transm..

[4] Kaarniranta K., Uusitalo H., Blasiak J., Felszeghy S., Kannan R., Kauppinen A., Salminen A., Sinha D., Ferrington D. (2020). Mechanisms of Mitochondrial Dysfunction and Their Impact on Age-Related Macular Degeneration. Prog. Retin. Eye Res..

[5] Naso F., Intartaglia D., Falanga D., Soldati C., Polishchuk E., Giamundo G., Tiberi P., Marrocco E., Scudieri P., Di Malta C. (2020). Light-responsive MicroRNA MiR-211 Targets Ezrin to Modulate Lysosomal Biogenesis and Retinal Cell Clearance. EMBO J..

[6] Kuo C., Green C.R., Rupenthal I.D., Mugisho O.O. (2020). Connexin43 Hemichannel Block Protects against Retinal Pigment Epithelial Cell Barrier Breakdown. Acta Diabetol..

[7] Blasiak J., Pawlowska E., Szczepanska J., Kaarniranta K. (2019). Interplay between Autophagy and the Ubiquitin-Proteasome System and Its Role in the Pathogenesis of Age-Related Macular Degeneration. Int. J. Mol. Sci..

[8] Kroemer G., Mariño G., Levine B. (2010). Autophagy and the Integrated Stress Response. Mol. Cell.

[9] Kim Y.C., Guan K.L. (2015). MTOR: A Pharmacologic Target for Autophagy Regulation. J. Clin. Investig..

[10] Wong P.M., Puente C., Ganley I.G., Jiang X. (2013). The ULK1 Complex Sensing Nutrient Signals for Autophagy Activation. Autophagy.

[11] Pesce N.A., Canovai A., Lardner E., Cammalleri M., Kvanta A., Andr H., Monte M.D. (2021). Autophagy Involvement in the Postnatal Development of the Rat Retina Noemi. Cells.

[12] Wesselborg S., Stork B. (2015). Autophagy Signal Transduction by ATG Proteins: From Hierarchies to Networks. Cell. Mol. Life Sci..

[13] Metlagel Z., Otomo C., Takaesu G., Otomo T. (2013). Structural Basis of ATG3 Recognition by the Autophagic Ubiquitin-like Protein ATG12. Proc. Natl. Acad. Sci. USA.

[14] Pyo J.O., Nah J., Jung Y.K. (2012). Molecules and Their Functions in Autophagy. Exp. Mol. Med..

[15] Simonsen A., Tooze S.A. (2009). Coordination of Membrane Events during Autophagy by Multiple Class III PI3-Kinase Complexes. J. Cell Biol..

[16] Wurzer B., Zaffagnini G., Fracchiolla D., Turco E., Abert C., Romanov J., Martens S. (2015). Oligomerization of P62 Allows for Selection of Ubiquitinated Cargo and Isolation Membrane during Selective Autophagy. eLife.

[17] Itakura E., Mizushima N. (2011). P62 Targeting to the Autophagosome Formation Site Requires Self-Oligomerization but Not LC3 Binding. J. Cell Biol..

[18] Fader C.M., Colombo M.I. (2009). Autophagy and Multivesicular Bodies: Two Closely Related Partners. Cell Death Differ..

[19] Saftig P., Klumperman J. (2009). Lysosome Biogenesis and Lysosomal Membrane Proteins: Trafficking Meets Function. Nat. Rev. Mol. Cell Biol..

[20] Anckar J., Sistonen L. (2011). Regulation of HSF1 Function in the Heat Stress Response: Implications in Aging and Disease. Annu. Rev. Biochem..

[21] Ciechanover A., Kwon Y.T. (2017). Protein Quality Control by Molecular Chaperones in Neurodegeneration. Front. Neurosci..

[22] Kaushik S., Cuervo A.M. (2012). Chaperones in Autophagy. Pharmacol. Res..

[23] Brader H.S., Young L.H.Y. (2016). Subthreshold Diode Micropulse Laser: A Review. Semin. Ophthalmol..

[24] Lavinsky D., Wang J., Huie P., Dalal R., Lee S.J., Lee D.Y., Palanker D. (2016). Nondamaging Retinal Laser Therapy: Rationale and Applications to the Macula. Investig. Ophthalmol. Vis. Sci..

[25] Sramek C., Mackanos M., Spitler R., Leung L.S., Nomoto H., Contag C.H., Palanker D. (2011). Non-Damaging Retinal Phototherapy: Dynamic Range of Heat Shock Protein Expression. Investig. Ophthalmol. Vis. Sci..

[26] Amirkavei M., Pitkänen M., Kaikkonen O., Kaarniranta K., André H., Koskelainen A. (2020). Induction of Heat Shock Protein 70 in Mouse RPE as an In Vivo Model of Transpupillary Thermal Stimulation. Int. J. Mol. Sci..

[27] She H., Li X., Yu W. (2006). Subthreshold Transpupillary Thermotherapy of the Retina and Experimental Choroidal Neovascularization in a Rat Model. Graefe’s Arch. Clin. Exp. Ophthalmol..

[28] Desmettre T., Maurage C.A., Mordon S. (2001). Heat Shock Protein Hyperexpression on Chorioretinal Layers after Transpupillary Thermotherapy. Investig. Ophthalmol. Vis. Sci..

[29] Wang J., Huie P., Dalal R., Lee S., Tan G., Lee D., Lavinksy D., Palanker D. (2016). Heat Shock Protein Expression as Guidance for the Therapeutic Window of Retinal Laser Therapy. Ophthalmic Technol. XXVI.

[30] Inagaki K., Shuo T., Katakura K., Ebihara N., Murakami A., Ohkoshi K. (2015). Sublethal Photothermal Stimulation with a Micropulse Laser Induces Heat Shock Protein Expression in ARPE-19 Cells. J. Ophthalmol..

[31] Kern K., Mertineit C.L., Brinkmann R., Miura Y. (2018). Expression of Heat Shock Protein 70 and Cell Death Kinetics after Different Thermal Impacts on Cultured Retinal Pigment Epithelial Cells. Exp. Eye Res..

[32] Mackanos M.A., Contag C.H. (2011). Pulse Duration Determines Levels of Hsp70 Induction in Tissues Following Laser Irradiation. J. Biomed. Opt..

[33] Viiri J., Amadio M., Marchesi N., Hyttinen J.M.T., Kivinen N., Sironen R., Rilla K., Akhtar S., Provenzani A., D’Agostino V.G. (2013). Autophagy Activation Clears ELAVL1/HuR-Mediated Accumulation of SQSTM1/P62 during Proteasomal Inhibition in Human Retinal Pigment Epithelial Cells. PLoS ONE.

[34] Golestaneh N., Chu Y., Xiao Y.Y., Stoleru G.L., Theos A.C. (2017). Dysfunctional Autophagy in RPE, a Contributing Factor in Age-Related Macular Degeneration. Cell Death Dis..

[35] Mitter S.K., Song C., Qi X., Mao H., Rao H., Akin D., Lewin A., Grant M., Dunn W., Ding J. (2014). Dysregulated Autophagy in the RPE Is Associated with Increased Susceptibility to Oxidative Stress and AMD. Autophagy.

[36] Dunn K.C., Aotaki-Keen A.E., Putkey F.R., Hjelmeland L.M. (1996). ARPE-19, a Human Retinal Pigment Epithelial Cell Line with Differentiated Properties. Exp. Eye Res..

[37] Hytti M., Korhonen E., Hongisto H., Kaarniranta K., Skottman H., Kauppinen A. (2021). Differential Expression of Inflammasome-Related Genes in Induced Pluripotent Stem-Cell-Derived Retinal Pigment Epithelial Cells with or without History of Age-Related Macular Degeneration. Int. J. Mol. Sci..

[38] Plaza Reyes A., Petrus-Reurer S., Padrell Sánchez S., Kumar P., Douagi I., Bartuma H., Aronsson M., Westman S., Lardner E., André H. (2020). Identification of Cell Surface Markers and Establishment of Monolayer Differentiation to Retinal Pigment Epithelial Cells. Nat. Commun..

[39] Sandqvist A., Björk J.K., Åkerfelt M., Chitikova Z., Grichine A., Vourc’h C., Jolly C., Salminen T.A., Nymalm Y., Sistonen L. (2009). Heterotrimerization of Heat-Shock Factors 1 and 2 Provides a Transcriptional Switch in Response to Distinct Stimuli. Mol. Biol. Cell.

[40] Sarge K.D., Murphy S.P., Morimoto R.I. (1993). Activation of Heat Shock Gene Transcription by Heat Shock Factor 1 Involves Oligomerization, Acquisition of DNA-Binding Activity, and Nuclear Localization and Can Occur in the Absence of Stress. Mol. Cell. Biol..

[41] Sistonen L., Sarge K.D., Morimoto R.I. (1994). Human Heat Shock Factors 1 and 2 Are Differentially Activated and Can Synergistically Induce Hsp70 Gene Transcription. Mol. Cell. Biol..

[42] Nivon M., Richet E., Codogno P., Arrigo A.P., Kretz-Remy C. (2009). Autophagy Activation by NFκB Is Essential for Cell Survival after Heat Shock. Autophagy.

[43] Lapierre L.R., Kumsta C., Sandri M., Ballabio A., Hansen M. (2015). Transcriptional and Epigenetic Regulation of Autophagy in Aging. Autophagy.

[44] Wiegant F.A.C., de Poot S.A.H., Boers-Trilles V.E., Schreij A.M.A. (2013). Hormesis and Cellular Quality Control: A Possible Explanation for the Molecular Mechanisms That Underlie the Benefits of Mild Stress. Dose-Response.

[45] Klionsky D.J., Abdel-Aziz A.K., Abdelfatah S., Abdellatif M., Abdoli A., Abel S., Abeliovich H., Abildgaard M.H., Abudu Y.P., Acevedo-Arozena A. (2021). Guidelines for the Use and Interpretation of Assays for Monitoring Autophagy (4th Edition). Autophagy.

[46] Rusmini P., Cristofani R., Galbiati M., Cicardi M.E., Meroni M., Ferrari V., Vezzoli G., Tedesco B., Messi E., Piccolella M. (2017). The Role of the Heat Shock Protein B8 (HSPB8) in Motoneuron Diseases. Front. Mol. Neurosci..

[47] Agholme L., Agnello M., Agostinis P., Aguirre-ghiso J.A., Ahn H.J., Ait-mohamed O., Brown E.J., Brumell J.H., Brunetti-pierri N., Brunk U.T. (2012). Guidelines for the Use and Interpretation of Assays for Monitoring Autophagy. Autophagy.

[48] Kivinen N., Hyttinen J., Viiri J. (2014). Hsp70 Binds Reversibly to Proteasome Inhibitor-Induced Protein Aggregates and Evades Autophagic Clearance in ARPE-19 Cells. J. Biochem..

[49] Mahat D.B., Salamanca H.H., Duarte F.M., Danko C.G., Lis J.T. (2016). Mammalian Heat Shock Response and Mechanisms Underlying Its Genome-Wide Transcriptional Regulation. Mol. Cell.

[50] Kmiecik S.W., Mayer M.P. (2022). Molecular Mechanisms of Heat Shock Factor 1 Regulation. Trends Biochem. Sci..

[51] Åkerfelt M., Morimoto R.I., Sistonen L. (2010). Heat Shock Factors: Integrators of Cell Stress, Development and Lifespan. Nat. Rev. Mol. Cell Biol..

[52] Batista-Nascimento L., Neef D.W., Liu P.C.C., Rodrigues-Pousada C., Thiele D.J. (2011). Deciphering Human Heat Shock Transcription Factor 1 Regulation via Post-Translational Modification in Yeast. PLoS ONE.

[53] Kumsta C., Chang J.T., Schmalz J., Hansen M. (2017). Hormetic Heat Stress and HSF-1 Induce Autophagy to Improve Survival and Proteostasis in C. Elegans. Nat. Commun..

[54] Komata T., Kanzawa T., Nashimoto T., Aoki H., Endo S., Nameta M., Takahashi H., Yamamoto T., Kondo S., Tanaka R. (2004). Mild Heat Shock Induces Autophagic Growth Arrest, but Not Apoptosis in U251-MG and U87-MG Human Malignant Glioma Cells. J. Neuro-Oncol..

[55] Oberley T.D., Swanlund J.M., Zhang H.J., Kregel K.C. (2008). Aging Results in Increased Autophagy of Mitochondria and Protein Nitration in Rat Hepatocytes Following Heat Stress. J. Histochem. Cytochem..

[56] Cuervo A.M., Wong E. (2012). Autophagy Gone Awry in Neurodegenerative Diseases. Nat. Neurosci..

[57] Barna J., Csermely P., Vellai T. (2018). Roles of Heat Shock Factor 1 beyond the Heat Shock Response. Cell. Mol. Life Sci..

[58] Trinklein N.D., Murray J.I., Hartman S.J., Botstein D., Myers R.M.M. (2004). The Role of Heat Shock Transcription Factor 1 in the Genome-Wide Regulation of the Mammalian Heat Shock Response. Mol. Biol. Cell.

[59] Zaffagnini G., Savova A., Danieli A., Romanov J., Tremel S., Ebner M., Peterbauer T., Sztacho M., Trapannone R., Tarafder A.K. (2018). P62 Filaments Capture and Present Ubiquitinated Cargos for Autophagy. EMBO J..

[60] Sun D., Wu R., Zheng J., Li P., Yu L. (2018). Polyubiquitin Chain-Induced P62 Phase Separation Drives Autophagic Cargo Segregation. Cell Res..

[61] Kageyama S., Gudmundsson S.R., Sou Y.S., Ichimura Y., Tamura N., Kazuno S., Ueno T., Miura Y., Noshiro D., Abe M. (2021). P62/SQSTM1-Droplet Serves as a Platform for Autophagosome Formation and Anti-Oxidative Stress Response. Nat. Commun..

[62] Dokladny K., Zuhl M.N., Mandell M., Bhattacharya D., Schneider S., Deretic V., Moseley P.L. (2013). Regulatory Coordination between Two Major Intracellular Homeostatic Systems: Heat Shock Response and Autophagy. J. Biol. Chem..

[63] Jin M., Klionsky D.J. (2009). Regulation of Autophagy: Modulation of the Size and Number of Autophagosomes. FEBS Lett..

[64] Xie Z., Nair U., Klionsky D.J. (2008). Atg8 Controls Phagophore Expansion during Autophagosome Formation. Mol. Biol. Cell.

[65] Kaikkonen O., Turunen T.T., Meller A., Ahlgren J., Koskelainen A. (2021). Retinal Temperature Determination Based on Photopic Porcine Electroretinogram. IEEE Trans. Biomed. Eng..

[66] Pitkänen M., Kaikkonen O., Koskelainen A. (2019). In Vivo Monitoring of Mouse Retinal Temperature by ERG Photoresponses. Exp. Eye Res..

[67] Pitkänen M., Kaikkonen O., Koskelainen A. (2017). A Novel Method for Mouse Retinal Temperature Determination Based on ERG Photoresponses. Ann. Biomed. Eng..

